# Enterohemorrhagic *Escherichia coli* pathogenesis: role of Long polar fimbriae in Peyer’s patches interactions

**DOI:** 10.1038/srep44655

**Published:** 2017-03-20

**Authors:** Charlotte Cordonnier, Lucie Etienne-Mesmin, Jonathan Thévenot, Amandine Rougeron, Sandra Rénier, Benoit Chassaing, Arlette Darfeuille-Michaud, Nicolas Barnich, Stéphanie Blanquet-Diot, Valérie Livrelli

**Affiliations:** 1 Université Clermont Auvergne, Inserm U1071, M2iSH “Microbes, Intestin, Inflammation et Susceptibilité de l’Hôte”, USC-INRA 2018, F-63000 Clermont-Ferrand, France; 2 Université Clermont Auvergne, MEDIS “Microbiologie Environnement DIgestif Santé”, F-63000 Clermont-Ferrand, France; 3 CHU Clermont-Ferrand, Service de Bactériologie, Parasitologie Mycologie, Clermont-Ferrand, F-63000, France

## Abstract

Enterohemorrhagic *Escherichia coli* (EHEC) are major food-borne pathogens whose survival and virulence in the human digestive tract remain unclear owing to paucity of relevant models. EHEC interact with the follicle-associated epithelium of Peyer’s patches of the distal ileum and translocate across the intestinal epithelium *via* M-cells, but the underlying molecular mechanisms are still unknown. Here, we investigated the involvement of Long polar fimbriae (Lpf) in EHEC pathogenesis. Of the 236 strains tested, a significant association was observed between the presence of *lpf* operons and pathogenicity. In sophisticated *in vitro* models of the human gastro-intestinal tract, *lpf* expression was induced during transit through the simulated stomach and small intestine, but not in the colonic compartment. To investigate the involvement of Lpf in EHEC pathogenesis, *lpf* isogenic mutants and their relative trans-complemented strains were generated. Translocation across M-cells, interactions with murine ileal biopsies containing Peyer’s patches and the number of hemorrhagic lesions were significantly reduced with the *lpf* mutants compared to the wild-type strain. Complementation of *lpf* mutants fully restored the wild-type phenotypes. Our results indicate that (i) EHEC might colonize the terminal ileum at the early stages of infection, (ii) Lpf are an important player in the interactions with Peyer’s patches and M-cells, and could contribute to intestinal colonization.

Enterohemorrhagic *Escherichia coli* (EHEC), as a subgroup of Shiga toxin (Stx)-producing *E. coli* (STEC), are food borne pathogens responsible for human diseases. In addition to uncomplicated diarrhea, EHEC can cause hemorrhagic colitis (HC) and life-threatening complications such as the hemolytic-uremic syndrome (HUS)^1^. Ruminants, especially cattle, are a natural reservoir of STEC, and human infection is linked with the consumption of contaminated food. STEC belong to a wide range of serotypes; however, only a limited number has been associated with human disease, among which EHEC O157:H7 is the most prevalent serotype associated with outbreaks and sporadic cases worldwide^2^. Based on the association of serotypes with diseases of varying severity in humans and with outbreaks or sporadic disease, STEC have been classified into 5 seropathotypes: A (associated with outbreaks and HUS and belonging to the O157:H7 serotype), B (associated with outbreaks and HUS but less commonly than serotype O157:H7), C (associated with sporadic HUS but not with outbreaks), D (associated with diarrhea but not with outbreaks or HUS cases), and E (serotypes that have not been involved in disease in humans)^3^. To date, the management of EHEC infections only includes supportive therapy, since antibiotics could worsen clinical outcomes^4^.

Survival and colonization of the human gastrointestinal (GI) tract are key features of EHEC infections but remain poorly described due to the lack of relevant models. EHEC viability and expression of virulence genes in the digestive environment have been mostly investigated in oversimplified *in vitro* models not representative of human physiological conditions^5^,^6^,^7^. In the human digestive tract, EHEC strains produce Shiga-toxins (Stx) considered to be essential for virulence and major risk factors for severe EHEC infections. Meanwhile, EHEC pathogenesis is not restricted to toxin-mediated effects, and a combination of virulence traits seems to be required, as demonstrated by the intimate bacterial attachment to host epithelial cells leading to the characteristic attaching and effacing (A/E) lesions^8^,^9^.

The terminal ileum and colon are considered to be the main sites of EHEC colonization in humans^10^. *In vitro* organ culture (IVOC) studies have demonstrated a preferential tropism of EHEC O157:H7 for the Follicle-Associated Epithelium (FAE) of Peyer’s patches, mainly localized in the distal ileum in humans^11^. It has been hypothesized that the Peyer’s patches-rich distal ileum might represent the initial site of EHEC adhesion and colonization, and EHEC would then spread to other regions of the gut^12^. Concurrently, the FAE promotes uptake of antigens and microorganisms through specialized epithelial cells with high transcytotic capacity, termed M-cells^13^,^14^. A previous study has suggested an interaction of EHEC strains with murine Peyer’s patches, followed by a translocation through M-cells from the gut lumen to underlying tissues. After bacterial uptake by M-cells, Stx induces apoptosis in underlying infected macrophages, which yields to toxin release in the *lamina propria*. Stx would then enter the bloodstream to reach target organs, leading to severe disease in humans^15^. However, underlying molecular mechanisms involved in M-cell targeting remain poorly investigated, and the bacterial effectors associated have not yet been defined.

Long polar fimbriae (Lpf), first described as putative adhesins in *Salmonella enterica* serovar Typhimurium, have been shown to attach directly to murine Peyer’s patches^16^,^17^. Lpf have also been previously described in Crohn’s disease-associated Adherent-Invasive *Escherichia coli* (AIEC) as key players in their interactions with M-cells^18^. Genome analysis of EHEC O157:H7 strain EDL933 revealed the presence of two *lpf* clusters encoded by O-islands 141 and 154, closely related to Lpf of *Salmonella* Typhimurium^19^,^20^,^21^. In EHEC O157:H7, Lpf have been reported to be involved in the adhesion process and micro-colony formation at the surface of cultured cells^21^,^22^ probably through binding to extracellular matrix protein^23^. Recent studies supported the role of Lpf in the induction of host pro-inflammatory responses to EHEC infection^24^,^25^. In EHEC O113:H21, a fimbrial cluster related to Lpf has also been identified located at the same position of the O island 154 in EHEC O157:H7 strain EDL933^26^. Lpf_OI-113_, identified in *E. coli* strains of other serogroups, may be involved in adherence of *E. coli* strains to epithelial cells^27^.

In the present study, the roles of Lpf in EHEC pathogenicity and in tropism to Peyer’s patches were investigated using *in vitro* and *in vivo* approaches. First, the prevalence of *lpf* operons was analyzed according to STEC seropathotype and origin, in a collection of 236 strains of known serotype. A strong association of the *lpf*_*OI-141*_ and *lpf*_*OI-154*_operons and pathogenicity was observed. Then, *lpf* expression was investigated in relevant dynamic *in vitro* models of the human digestive tract, with *lpf* being overexpressed in gastric and small intestinal conditions but not in colonic ones. Using *lpf* isogenic mutants and their relative trans-complemented strains, we showed that expression of *lpf* genes is required for an active translocation across M-cell monolayer *in vitro* and for interactions with Peyer’s patches in mice ileal loops *in vivo*.

## Results

### *lpfA*_*OI-141*_ and *lpfA*_*OI-154*_, but not *lpfA*_*OI-113*_, are associated with pathogenic strains

**Figure 1:**
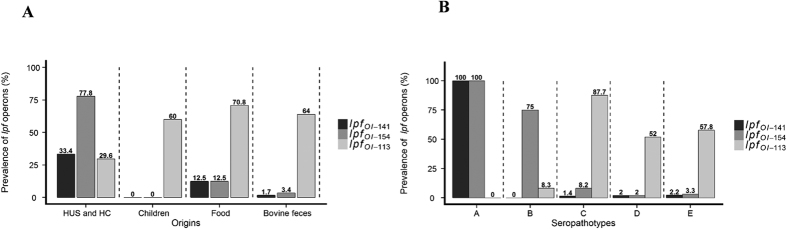
*lpf*_OI-141_ and *lpf*_OI-154_ are associated with the seropathotype A including the most virulent strains. (**A**,**B**) The prevalence of *lpf*_OI-141_, *lpf*_OI-154_ and *lpf*_OI-113_ operons was determined by PCR in a total of 236 STEC/EHEC isolates, and expressed as percentages of the total number of strains according to the strain origin (**A**) and seropathotype (**B**).

Some STEC serotypes recovered from animals or food have never been associated with human diseases, therefore STEC strains were classified into seropathotypes, according to association with HUS and outbreaks^28^. To assess the potential links between *lpf* operons and STEC pathogenicity, the prevalence of *lpfA*_*OI-141*_, *lpfA*_*OI-154*_ and *lpfA*_*OI-113*_ was investigated in a collection of 236 STEC strains isolated from humans, animals or food and classified according to the source and seropathotype (Fig. 1). Among the 236 strains of known serotype^29^,^30^, 175 were isolated from bovine feces, 27 from HUS and HC patients, 24 from food samples and 10 from asymptomatic children. The *lpf*_*OI-141*_and *lpf*_*OI-154*_ operons were identified in 15 (6.4%) and 30 (12.7%) of the 236 STEC strains, respectively, *versus* 143 (60.6%) for *lpfA*_*OI-113*_ (Supplemental Fig. 1A and B). However, *lpf*_*OI-141*_ and *lpf*_*OI-154*_ were significantly (p < 0.01) more prevalent in strains isolated from HUS and HC patients than from other sources (Fig. 1A), while *lpfA*_*OI-113*_ was mainly found in strains isolated from bovine feces (112 versus 8 in HUS and HC patients, p < 0.001). When the strains were categorized according to seropathotype (Fig. 1B), the prevalence of *lpf*_*OI-141*_ and *lpf*_*OI-154*_was significantly higher (p < 0.001) and reached 100% in the seropathotype A (which includes the most virulent strains linked with severe disease and outbreaks), compared to other seropathotypes. By contrast, the prevalence of *lpf*_*OI-113*_ was significantly higher (p < 0.001) in seropathotypes C to E (no link with disease) than in seropathotypes A and B. All the strains belonging to seropathotype A harbored both *lpf*_*OI-141*_ and *lpf*_*OI-154*_ operons, but not the *lpf*_*OI-113*_genes (Fig. 1B). Overall, a strong association was observed between STEC strains harboring both *lpf*_*OI-141*_ and *lpf*_*OI-154,*_and belonging to seropathotype A, including O157:H7 strains known to be involved in the majority of human cases.

### *lpf* operon expression is induced *in vitro* in the stomach and small intestine but not in the large intestinal compartment

**Figure 2:**
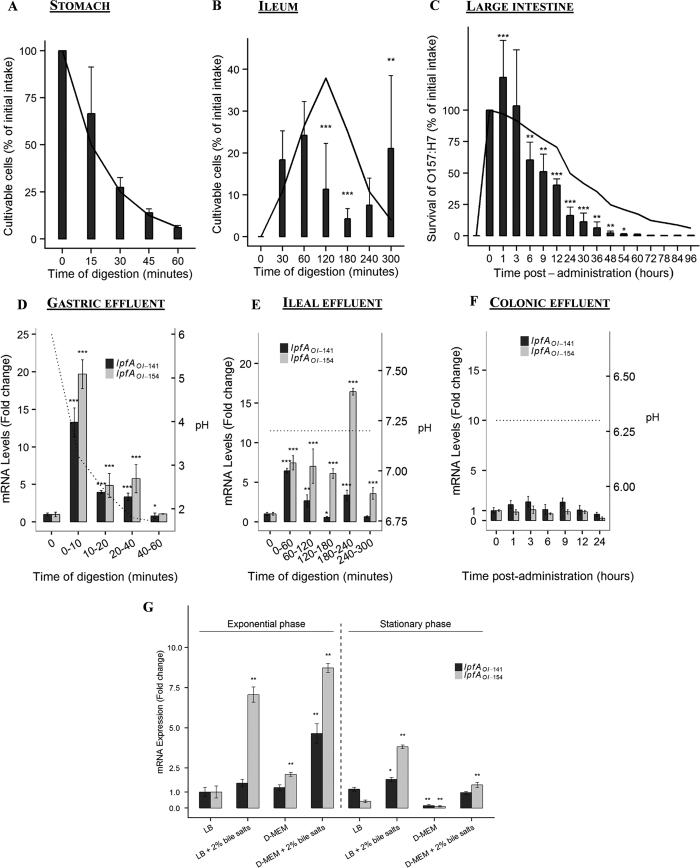
*lpf* genes are expressed in the human simulated gastric and small intestine environment but not in the colon. EHEC O157:H7 strain EDL933 survival and *lpf* gene expression were assessed in the artificial stomach (**A** and **D**), ileum (**B** and **E**) and colon **(C** and **F)**, after administration of 10^7^ UFC/mL of EHEC O157:H7 strain EDL933. **(A,B** and **C)** The bacterial population was estimated by regular plating on LB medium or by qPCR on *stx1* gene, in the TNO GastroIntestinal model (TIM) model (stomach, ileum) and Artificial COLon (ARCOL) model (large intestine), respectively. The profiles obtained for EDL933 were compared to that of theoretical transit markers (black line). Results are expressed as percentages of initial intake ± standard deviations (n = 3). Results obtained for EDL933 significantly different from that of the transit marker at p < 0.05 (*), p < 0.01 (**) and p < 0.001 (***). **(D**,**E** and **F)** Total RNAs were extracted from the gastric and ileal effluents of the TIM model and from the colonic medium in ARCOL and *lpfA*_*OI-141*_ and *lpfA*_*OI-154*_ expression was analyzed by RTq-PCR. The black dotted line indicates pH in the different digestive compartments. The 16S rRNA gene was used as an internal standard to normalize the data. Results are expressed as means of fold-induction (calculated using the Ct method with t0 as reference) ±standard deviations (n = 3). Time points statistically different from t0 at p < 0.05 (*), p < 0.01 (**) and p < 0.001 (***). (**G**) Influence of growth phase and bile salts (2%) on the expression of *lpfA*_*OI-141*_ and *lpfA*_*OI-154*_ was investigated in LB and DMEM media. Total RNAs were extracted after 3 h (exponential phase) or overnight (stationary phase) from a culture of EHEC O157:H7 strain EDL933 and *lpfA*_*OI-141*_ and *lpfA*_*OI-154*_ expression was analyzed by RTq-PCR. Results are expressed as means of fold-induction (calculated using the Ct method with t0 as reference) ±standard deviations (n = 3). Statistically different from results obtained in LB medium without bile salts during the exponential phase at p < 0.05 (*) or p < 0.01 (**).

Survival kinetics of *E. coli* O157:H7 strain EDL933 were investigated in TIM (TNO GastroIntestinal Model) and ARCOL (Artificial COLon) models (Fig. 2A–C). The TIM model simulates the stomach, and the three segments of the small intestine and the ARCOL system mimics the large intestine environment. In these *in vitro* models, results were expressed as percentages of initial intake and cross-compared to those obtained with a theoretical transit marker giving a 100% survival rate for bacteria. Bacterial counts below that of the transit marker will reflect cell mortality, while counts above the transit marker will be an indicator of bacterial growth. During gastric transit, no significant difference was observed between the curve obtained for bacteria and that of the transit marker, indicating that the viability of EDL933 was not modified by gastric conditions (Fig. 2A). Meanwhile, in the ileal compartment (Fig. 2B), a significant (p < 0.001) bacterial mortality was observed between 120 and 180 min. At 180 min, bacterial recovery percentages were 4.3 ± 2.4% (n = 3) compared to 25.2% for the transit marker. The trend reversed at the end of digestion in the ileal compartment (300 min) when the curve for bacteria exceeded that of the transit marker, indicating a bacterial growth with a survival percentage of 21.1 ± 17.4% (n = 4) for EDL933 compared to 4.0% for the transit marker. In ARCOL (Fig. 2C), even if a bacterial growth was observed 1 h post-administration (126.1 ± 29.4% for bacteria *versus* 97.1% for the marker, p < 0.001), from 6 h to the end of the experiment, we observed that EDL933 was eliminated from the colonic medium at a rate more rapid than that of the theoretical transit marker. These results indicate an absence of colonization in the bioreactor.

The *lpfA*_*OI-141*_and *lpfA*_*OI-154*_expression levels were then measured in the *in vitro* models (Fig. 2D–F). In the gastric effluents from the TIM system, *lpfA*_*OI-141*_and *lpfA*_*OI-154*_were over-expressed whatever the sampling time (Fig. 2D): the highest expression levels were observed at 0–10 min, with fold increases of 13.3 ± 4.7% and 19.7 ± 4.7% (n = 3, p < 0.001) for *lpfA*_*OI-141*_and *lpfA*_*OI-154*_respectively, compared to the initial time point. In the ileal effluents, a similar trend was observed from 60 to 300 min, with up to 6.4 and 16.4 fold induction of *lpfA*_*OI-141*_ and of *lpfA*_*OI-154*_ respectively (p < 0.05) (Fig. 2E). In contrast, *lpf* expression was not significantly induced in the colonic medium whatever the sampling time assayed (Fig. 2F).

To better understand the parameters of human digestion controlling *lpf* genes expression, we further assessed the effect of bile salts on *lpf*A_OI-141_ and *lpf*A_OI-154_ mRNA levels when *E. coli* O157:H7 strain EDL933 was grown until exponential or stationary phases in Luria Bertani (LB) or cell culture medium (DMEM) (Fig. 2G). In the exponential phase, *lpfA*_*OI-154*_ expression levels were significantly higher (p < 0.01) in DMEM compared to LB medium. Also, bile salts induced an increase in *lpfA*_*OI-141*_ and *lpfA*_*OI-154*_ expression when bacteria were either in exponential or stationary growth phases. The highest expression levels were observed with *lpfA*_*OI-154*_ when grown to exponential phase, in both LB and DMEM media, with up to 9 fold increase (p < 0.05).

### Type I pili are not expressed in EHEC O157:H7 EDL933

**Figure 3:**
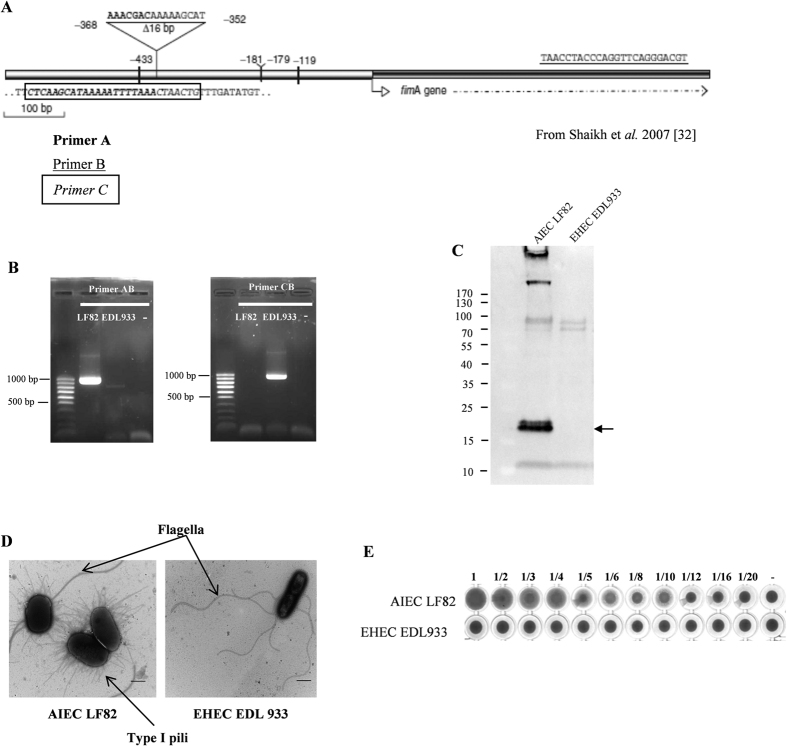
Absence of type I pili expression in EHEC O157:H7 strain EDL933. (**A**) Schematic representation of the PCR strategy described by Shaikh et *al.*^32^ to show the deletion (Δ16 bp) in the Fim region in *E. coli* O157:H7 strains. Primers A (bolded nucleotides) and B (underlined nucleotides) produce an amplicon in an intact configuration. Primers C (italicized nucleotides in box) and B produce an amplicon in a deleted configuration. (**B**) Ethidium bromide staining showing detection in the Fim region of EHEC O157:H7 strain EDL933 and AIEC strain LF82 (positive control). (**C**) Western blot analysis of type I pili in EHEC O157:H7 strain EDL933 and AIEC strain LF82 using the anti-αF1 antibody. The predicted size of FimA is 18.2 kDa (indicated by arrow). (**D**) Transmission electron micrographs of negatively stained EHEC O157:H7 strain EDL933 and AIEC strain LF82 strains taken at ×15,000 magnification (scale bar = 5 μm). Type I pili and flagella are indicated by arrows. (**E**) Results of yeast agglutination assays with EHEC O157:H7 strain EDL933 and AIEC strain LF82. Dilution rates of bacterial suspension are indicated on the corresponding wells. Negative control without bacteria (−).

*E. coli* bacteria were previously reported to interact with Peyer’s patches via type I pili^31^. Since Shaikh *et al*.^32^ showed that a deletion in the *fim* operon results in the absence of type I pili expression in most O157:H7 strains, we next investigated the type I pili genotype in EHEC O157:H7 strain EDL933 using the PCR strategy previously described (Supplemental Methods; Fig. 3A). Primers C and B produced a 936 pb amplicon with O157:H7 strain EDL933, whereas no amplification was observed with primers A and B, indicating a deletion in the *fim* operon in this strain (Fig. 3B). By contrast, this deletion is absent in the control AIEC strain LF82. Western blot analysis using polyclonal anti-αF1 antibody (Fig. 3C) and electron microscopic visualization of negatively stained bacteria (Fig. 3D) confirmed the absence of type I pili on the surface of O157:H7 strain EDL933, while AIEC strain LF82 harbored those pili. Finally, since type I pili expression can be appreciated by yeast agglutination (Supplemental Methods), we also investigated the capacity to agglutinate of EDL933 and LF82 with yeast. While visible agglutinates were observed with the positive control (LF82 strain) until 1/10 dilution, EDL933 did not agglutinate (Fig. 3E).

### Deletion of *lpf* genes does not alter bacterial growth, mobility nor cytotoxicity

To investigate the involvement of Lpf in the ability of EHEC O157:H7 strain EDL933 to target Peyer’s patches, Δ*lpfA*_*OI-141*_, Δ*lpfA*_*OI-154*_, Δ*lpfA*_*OI-141*_-Δ*lpfA*_*OI-154*_ isogenic mutants and mutants trans-complemented with their respective *lpf* genes were generated. We also used an O6:H10 strain (NV110) naturally deficient in *lpf* genes as previously reported by Pradel *et al*.^33^. O6:H10 strains are widely found in cattle but have never been involved in human infections^29^,^33^. The growth rate and the motility of wild-type strain EDL933, the *lpf* isogenic mutants and the trans-complemented strains do not markedly differ (Supplemental Fig. 2A,B), indicating that *lpf* genes are not essential for bacterial growth and motility. Electron microscopic observation confirmed that deletion of *lpf* genes does not alter their motility, or flagella expression (Supplemental Fig. 2C). Also, *lpf* deletion does not alter Stx production (Supplemental Fig. 2D) since Stx titers were similar for the wild-type EDL933 strain (cytotoxic titer 1/256 equivalent to 8.2 ± 1.8 ng/ml).

### Deletion of *lpf* genes does not modify EHEC adhesion to Caco-2 immortalized epithelial cells

Since Lpf were previously described to interact with intestinal epithelial cells, adhesion capacity of EDL933 wild-type strain, isogenic mutants and trans-complemented strains was investigated using the Caco-2 human colonic carcinoma cell line (Supplemental Fig. 2E). Bacterial strains were cultivated in conditions enhancing *lpf* expression (i.e. exponential phase, in the presence of bile salts). In the conditions tested, all EHEC strains adhered to Caco-2 cells at a very low level compared to AIEC strain LF82 (<5% versus 22.2 ± 7.3%) (p < 0.001). No significant difference was observed between the wild-type EDL933 strain (0.6 ± 0.2%), the Δ*lpfA*_*OI-141*_/Δ*lpfA*_*OI-154*_ isogenic mutant (0.9 ± 0.7%) or the trans-complemented strain (1.3 ± 1.6%), indicating that Lpf are not involved in EHEC adhesion to Caco-2 epithelial cells *in vitro*.

### Lpf are required for an active translocation of EHEC across human M-cells monolayer *in vitro*

**Figure 4:**
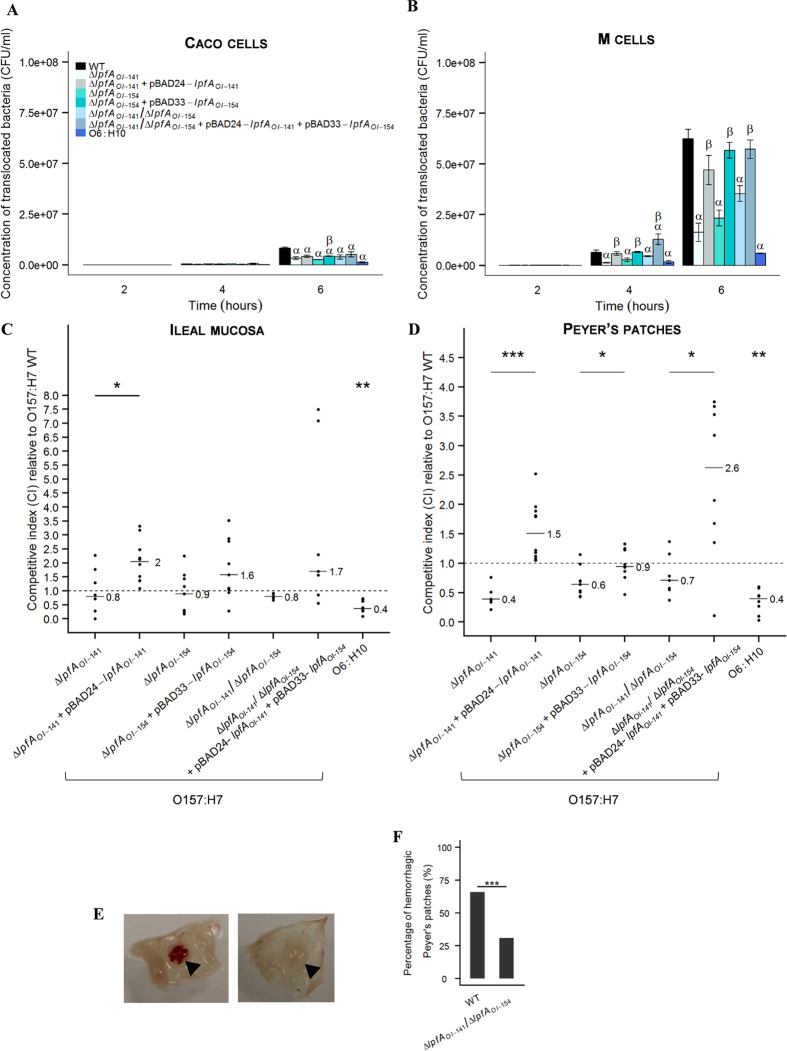
Lpf are needed for active translocation of EHEC strain EDL933 across M-cell monolayers and *in vivo* interactions with murine Peyer’s patches. Caco-2 cl1 and M-cells were infected with 10^7^ EHEC bacteria (wild-type EHEC O157:H7 strain EDL933, *lpf* isogenic mutants, trans-complemented strains and O6:H10 strain NV110) per Transwell^TM^. The number of translocated bacteria across Caco-2 cl1 (**A**) and M-cells (**B**) was determined at 2, 4 and 6 h post-infection by plating on selective agar plates. Results are expressed as mean CFU/ml ± standard deviation (n = 3). Translocation through Caco-2 cl1 or M-cells significantly different between wild-type strain EDL933 and isogenic mutants or O6:H10 NV110 at p < 0.001 (α). Translocation through Caco-2 cl1 or M-cells significantly different between isogenic mutants and trans-complemented strains at p < 0.001 (β). (**C** and **D**) Mice ileal loops (n = 10) were inoculated with a mixed inoculum containing equivalent number (5∙10^8^ CFU/ml) of EHEC O157:H7 EDL933 wild-type strain and either *lpf* isogenic mutants, trans-complemented mutants or the O6:H10 strain NV110. Bacterial interactions with ileal mucosa (**C**) or Peyer’s patches (**D**) were determined by competitive index (CI) analysis relative to the O157:H7 wild-type strain. Significant differences between CI obtained with isogenic mutants and the trans-complemented strains at p < 0.05 (*), p < 0.001 (**) or p < 0.001 (***). CI of O6:H10 significantly different from 1 at p < 0.001 (**). (**E**) Macroscopic view of hemorrhagic (*left*) or non-hemorrhagic (*right*) Peyer’s patches. (**F**) The percentages of hemorrhagic Peyer’s patches were determined in mice ileal loops infected with wild-type EHEC strain EDL933 (N = 32) or Δ*lpfA*_*OI-141*_/Δ*lpfA*_*OI-154*_ isogenic mutant (N = 26). Percentages obtained with Δ*lpfA*_*OI-141*_/Δ*lpfA*_*OI-154*_mutant significantly different from that of wild-type strain at p < 0.001 (***).

Using polarized enterocyte-like Caco-2 cl1 cells that acquire M-cell-like characteristics when co-cultured with the Raji B cell line, the interactions of EHEC with M-cells were investigated and compared to Caco-2 cl1 monocultures. Whereas high levels of translocation were observed across M-cell monolayers for EDL933, only a few bacteria translocated across Caco-2 cl1 monolayers after 6 h of infection (Fig. 4A and B). The translocation of EHEC bacteria across Caco-2 cl1 or M-cells was not the result of a loss of the monolayer integrity since TEER stayed constant during the 6 h of infection (data not shown). At 4 and 6 h postinfection, a significant (p < 0.001) decrease in the amount of translocated bacteria across M-like cells was observed for Δ*lpfA*_*OI-141*_, Δ*lpfA*_*OI-154*_, and Δ*lpfA*_*OI-141*_/Δ*lpfA*_*OI-154*_ mutants compared to the wild-type strain (Fig. 4B). Trans-complementation of *lpfA*_*OI-141*_ and/or *lpfA*_*OI-154*_ genes restored the ability of mutant strains to translocate specifically across M-cells (Fig. 4B). Interestingly, after a 6 h infection EHEC O6:H10, which is naturally deficient in *lpf* genes, also translocated at levels drastically lower (p < 0.001) compared to those of the wild-type O157:H7 strain. Altogether, these results indicate that Lpf play a key role in targeting M-cells.

### Lpf are needed for EHEC interactions with murine Peyer’s patches *in vivo*

The interaction between *lpf* isogenic mutants and murine Peyer’s patches was analyzed *in vivo* in a competitive assay in ileal loop where a mixed inoculum comprising equivalent numbers of two bacterial strains (wild-type strains and either *lpf* isogenic mutants, trans-complemented strains or a strain naturally deficient in *lpf* genes) is inoculated into ligated ileal loop. At 5 h post infection, the numbers of mucosa-interacting bacteria were counted to define competitive indices (CI) relative to the O157:H7 wild-type strain. CI close to 1 indicated a similar level of interactions with intestinal tissue (ileal mucosa or Peyer’s patches) between the two strains tested. In ileal mucosa, CIs close to 1 were observed when wild-type EDL933 was co-inoculated with Δ*lpfA*_*OI-141*_ (0.8 ± 0.8), Δ*lpfA*_*OI-154*_ (0.9 ± 0.7) or Δ*lpfA*_*OI-141*_*/*Δ*lpfA*_*OI-154*_ (0.8 ± 0.1) isogenic mutants (Fig. 4C) indicating that *lpf* genes have no influence on the interactions of EHEC EDL933 with ileal mucosa. By contrast, significant decreases in CI were observed in Peyer’s patches when Δ*lpfA*_*OI-141*_ (0.4 ± 0.2), Δ*lpfA*_*OI-154*_ (0.6 ± 0.2) and Δ*lpfA*_*OI-141*_*/*Δ*lpfA*_*OI-154*_ (0.7 ± 0.4) isogenic mutants were co-inoculated with wild-type strain (Fig. 4D), indicating that deletion of *lpf* genes significantly reduced the ability of O157:H7 EDL933 to interact with Peyer’s patches. Trans-complementation of *lpf* mutants restored their ability to interact with Peyer’s patches, at a level similar to that of the wild-type strain for Δ*lpfA*_*OI-154*_ (0.9 ± 0.3) or even higher for Δ*lpfA*_*OI-141*_ (1.5 ± 0.5) and Δ*lpfA*_*OI-141*_*/*Δ*lpfA*_*OI-154*_ (2.6 ± 1.3). These results were confirmed with an O6:H10 strain (lacking *lpf* genes) which showed a CI of 0.4 ± 0.1, close to that of *lpf* mutants. Our data indicate that Lpf are required for EHEC interactions with Peyer’s patches. Bloodshot Peyer’s patches were macroscopically observed following infection with wild-type EDL933 strain or Δ*lpfA*_*OI-141*_*/*Δ*lpfA*_*OI-154*_mutants (Fig. 4E). A significant (p < 0.001) decrease in the number of hemorrhagic Peyer’s patches was observed with the double mutant compared to the wild-type strain (31 versus 66%; Fig. 4F).

## Discussion

Food-borne infections caused by EHEC bacteria have emerged as an important public health concern worldwide. EHEC survival and virulence in the human digestive tract, as well as interactions with the intestinal epithelium, are key features in bacterial pathogenesis but remain largely unknown. It is well documented that several pathogenic microorganisms use M-cells and Peyer’s patches to invade the host intestinal mucosa^31^.

Up to date, few studies have investigated the behavior of EHEC strains in human simulated GI conditions. Studies available have been carried out in oversimplified *in vitro* systems^5^,^6^,^7^ integrating only a limited number of digestive parameters (such as gastric acidic pH or bile salts), far from *in vivo* complexity. Using the dynamic TIM model that reproduces human GI digestive process, we demonstrated that EDL933 survival was not affected by human-simulated gastric conditions when administered with water. EHEC are considered as acid resistant bacteria, but large variations in survival rates have been shown for *E. coli* O157:H7 in acidified culture media^35^ or in simulated gastric fluid (SGF)^36^,^37^,^38^. This heterogeneity may be explained by differences in culture conditions, bacterial strains and pH values used to simulate the gastric phase. Regarding the ileal compartment, we observed a loss of viability after a 2-h period of digestion, whereas a bacterial growth occurred in the late phase (i.e. after 240 min). Similar results have been previously obtained when EHEC bacteria were inoculated within food matrices in the TIM model^38^, ^39^, ^40^. Bacterial growth at the end of digestion could be attributed to lower concentrations of bile salts due to their passive reabsorption. Finally, in the simulated colonic conditions, EDL933 was not able to colonize, probably through the barrier effect of gut microbiota, as previously shown by Duncan *et al*.^41^ and by recent studies from the laboratory^42^,^43^. Altogether, our results suggest a potential ability of EHEC to colonize the human gut through a mechanism involving an important growth of the pathogen in the distal parts of the small intestine. Bacterial survival in the stomach, followed by growth in the distal compartments of the small intestine might account for the very low infectious dose reported for EHEC^1^.

EHEC strains must not only survive in the human GI tract but also orchestrate a complex machinery including expression of virulence determinants in response to localized gut microenvironments. Stx is known to be the main virulence trait of EHEC responsible for systemic complications, but the pathogen produces also a large number of proteins, which contribute to its establishment, persistence and tissue tropism. Colonization factors such as Long polar fimbriae (Lpf) are found to be involved in the key steps of EHEC pathogenesis, such as adhesion, translocation or inflammation^44^,^45^. Genome analysis of EHEC O157:H7 reference strain EDL933 revealed the presence of two *lpf* operons, *lpfA*_*OI-141*_ and *lpfA*_*OI-154*_, also named *lpf1* and *lpf2*^21^. A third operon, *lpfA*_*OI-113*_ has been identified in O113:H21 EHEC strains, commonly associated with sporadic cases of infection^26^. We investigated by PCR the prevalence of *lpfA*_*OI-141*_, *lpfA*_*OI-154*_ and *lpfA*_*OI-113*_ among a wide range of strains (n = 236) isolated from bovine feces, food and humans. We showed that *lpfA*_*OI-141*_and *lpfA*_*OI-154*_ were more prevalent in strains isolated from HUS patients, whereas *lpfA*_*OI-113*_ was mainly associated with strains isolated from bovine feces. Our results are in accordance with those of others^19^,^20^,^21^ who reported that *lpfA*_*OI-141*_ and *lpfA*_*OI-154*_ were more prevalent in human samples and that the *lpf* gene was significantly associated with HUS. Herein, the analysis of the 236 strains (classified into the five seropathotypes according to association with severe diseases) revealed a positive correlation between the presence of *lpfA*_*OI-141*_ and *lpfA*_*OI-154*_ and seropathotype A (involved in severe human cases)^46^, while *lpfA*_*OI-113*_ was significantly associated with seropathotypes C to E (no link with disease). These data, in accordance with studies conducted by others^47^,^48^, strengthen the role for *lpfA*_*OI-141*_ and *lpfA*_*OI-154*_ in human infection.

**Figure 5:**
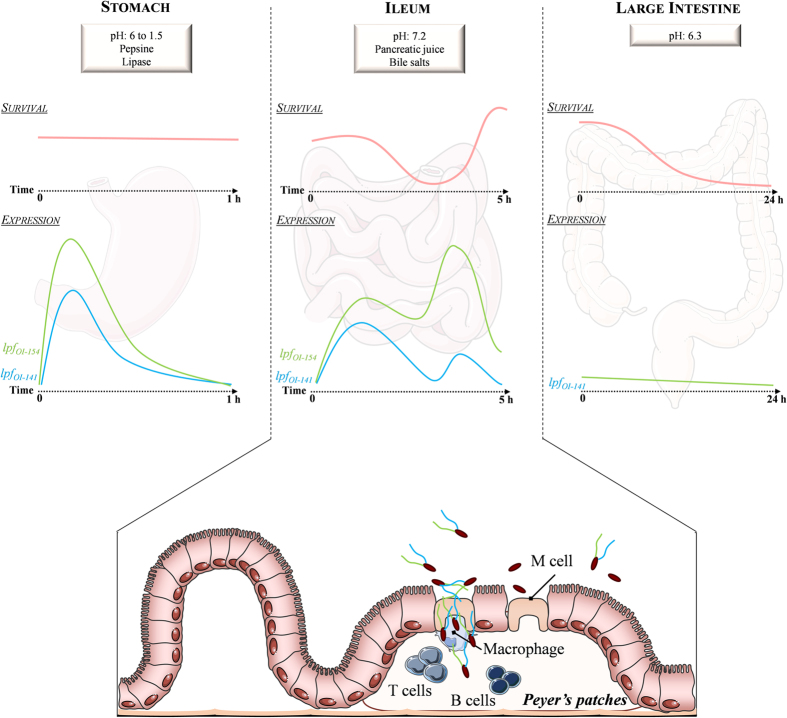
Proposed role of Lpf in EHEC pathogenicity. The figure summarizes the main results obtained from this study. The survival rate of EHEC is shown in red. In the gastric compartment EHEC can survive; while in the small intestinal compartments, mortality is followed by a bacterial growth at the end of digestion, in the large intestine, the pathogenic bacteria are not able to maintain and are progressively eliminated. The *lpfA*_*OI-141*_ and *lpfA*_*OI-154*_gene expression profiles, analyzed throughout the simulated human digestive tract, are shown in blue and green respectively. *lpf* expression is increased during bacterial transit through the gastric and small intestine, mainly in the terminal ileum. Gastric pH and bile salts concentration in small intestine are hypothesized to be the factors inducing *lpf* expression. The absence of Lpf impairs EHEC ability to interact with ileal Peyer’s patches. Our results suggest that EHEC might colonize the terminal ileum at the early stages of infection in humans, and that Lpf are involved in the interactions of EHEC with ileal Peyer’s patches and are needed for an active translocation of the pathogen across M-cells.

To date, the regulation of EHEC *lpf* genes in the human digestive environment was only investigated in simplistic *in vitro* approaches. In our complete and sophisticated *in vitro* GI and gut models, *lpfA*_*OI-141*_ and *lpfA*_*OI-154*_were highly expressed in the gastric and small intestinal environment, but not in the colon (Fig. 5). Here, we showed that in the gastric effluents, *lpfA*_*OI-141*_ and *lpfA*_*OI-154*_were over-expressed at the beginning of digestion (from t0 to 10 min) when the pH was between 6 and 3. In the ileal effluents (pH 7.2), both *lpf* genes were expressed throughout digestion, being highly expressed at the end of the experiment simultaneously with bacterial growth. These results, obtained in dynamic models closely mimicking human digestion, confirm that the exponential growth phase, body temperature (37 °C) and pH close to the neutrality (pH 6.5) favor *lpf* expression^49^. To further investigate the parameters influencing *lpf* expression in the human GI tract, we appreciated the effect of bile salts in batch cultures. As previously observed by others^50^,^51^, we demonstrated that *lpf* mRNA levels exhibited a significant increase when bile salts were added in the media. Upregulation by bile salts could explain the significant increase of *lpf* mRNA levels in the distal small intestine (and not in the colon), associated with higher concentrations of bile salts in the ileum. In batch cultures, we demonstrated that *lpfA*_*OI-154*_mRNA levels are significantly higher compared to *lpfA*_*OI-141*_ in the presence of bile salts. This corroborates the results obtained in ligated pig intestine, where bile salts increased *lpf2* but not *lpf1* expression^50^. In other pathogenic *E. coli* bacteria such as AIEC, bile salts have already been described as an activator of *lpf* transcription in a mechanism related to a transcriptional regulator, FhlA, only present in enteric bacteria^51^. In *E. coli* O157:H7, it has been shown that *lpf* operon expression is tightly controlled by global regulators such as histone-like nucleoid-structuring protein (H-NS) and LEE-encoded regulators^52^,^53^, emphasizing the complexity of *lpf* genes regulation. Our data suggest that the ileum could be an important site for expression of EHEC adhesins such as Lpf, which genes can be induced even without direct contact with the host cells. This also indicates that EHEC might colonize this segment of the gut at the early stages of infection in humans.

Once EHEC bacteria have crossed the gastric and small intestinal barriers, they intimately interact with the host epithelium. We assessed the role of *lpfA*_*OI-141*_ and *lpfA*_*OI-154*_ in EHEC adhesion to intestinal cells in culture. In the conditions tested, O157:H7 EDL933 only slightly adhered to Caco-2 cells (<5% of bacterial inoculum) through a mechanism independent of *lpf* expression. With regard to the role of Lpf in bacterial adhesion, conflicting results are reported. Deletion of *lpfA*_*OI-141*_ and/or *lpfA*_*OI-154*_genes led to a decrease in adhesion to epithelial cells^21^,^22^,^23^,^27^,^54^,^55^, but this effect was observed *in vitro* only at early time points (at 3 h but not at 5 or 6 h), suggesting a role of *lpf* in the early stage of adhesion^21^,^22^. *In vivo* in several animal models (rabbit, lamb, sheep, and pig), *lpfA*_*OI-141*_ and *lpfA*_*OI-154*_ mutants were recovered at significantly lower levels in the feces compared to the wild-type strain, showing a role of Lpf in EHEC O157:H7 colonization^49^,^56^,^57^. However, *lpf*2 did not influence the ability of EHEC O157:H7 strain 86–24 to adhere to intestinal explants obtained from lambs^49^. In addition, studies from other laboratories working on enteric pathogens such as *Citrobacter rodentium* (natural mouse pathogen used as a model for studying EHEC virulence), AIEC or *Salmonella* have shown that Lpf are not involved in the adhesion to various epithelial cells^18^,^58^,^59^.

Apart from adhesion to epithelial cells, EHEC can also interact with Peyer’s patches in the host epithelium. Using IVOC of human intestinal tissue, EHEC demonstrated a particular tropism for the FAE of the distal ileal Peyer’s patches rich in M-cells^11^,^55^. In addition to EHEC, fourteen different species of pathogenic and non-pathogenic bacteria selectively adhere to and exploit the M-cell transport mechanism to infect mucosal tissues and/or spread systemically^60^,^61^. The mechanisms of interactions with M-cells involve invasin and internalin B, respectively in *Yersinia pseudotuberculosis*^62^ and *Listeria monocytogenes*^63^. Recently, *E. coli* were reported to interact with Peyer’s patches via the recognition by type I pili of glycoprotein 2 (GP2), which is specifically localized on apical plasma membrane of M-cells^31^. Previous studies have shown that type I fimbriae are not expressed in strains of *E. coli* serotype O157 : H7 because of a 16 bp deletion in the *fimA* regulatory region^32^,^64^. Such deletion was confirmed in the present study and lead us to speculate that adhesins other than type I pili are involved in EHEC tropism to M-cells.

Here, we have shown by using complementary *in vitro* human M-cell model and *in vivo* mice ileal loops, that *lpfA*_*OI-141*_ and *lpfA*_*OI-154*_mediate EHEC interactions with Peyer’s patches, leading to a significant reduction in the number of hemorrhagic Peyer’s patches with *lpf* mutants compared to the wild-type strain. The role of EHEC Lpf in targeting Peyer’s patches was further supported by the results obtained with the O6:H10 NV110 strain, which constitutively lacks *lpf* genes. The capacity of O6:H10 to interact with specialized M-cells was significantly reduced compared to O157:H7 strain EDL933, whereas similar adhesion levels to intestinal epithelial cells were found for both strains. Lpf have also been identified as a key factor for *Salmonella* Typhimurium and AIEC in targeting Peyer’s patches^18^,^65^. We found that the trans-complementation of the double mutant led to a significant increase in EDL933 interactions with Peyer’s patches compared to the wild-type strain. This could be linked to an overexpression of *lpf* genes or to a deregulation of other factors. Lloyd *et al*.^57^ observed that the *lpf1 lpf2* double mutant strain of O157:H7 86–24 showed an increased adhesion to Caco-2 cells compared to the parent strain, which resulted from an over-expression of curli on the bacterial surface. Although we did not observe such phenotype at the bacterial surface, this suggests that the regulatory pathways governing the expression of *lpf* and other fimbriae are inter-related. Advances have to be made in establishing the host binding partner for Lpf. Previous studies demonstrated *in vitro* that Lpf are involved in the binding of EHEC to extracellular matrix proteins, increasing the number of adherent EHEC bacteria to cultured intestinal cells^23^,^66^. Such interactions might enhance colonization at sites where the mucosal barrier is injured by Shiga-toxins. Our results suggest that a specific receptor for Lpf may be expressed at the surface of Peyer’s patches and M-cells. These specific receptors have not been identified to date. Such investigations are hampered by the failure to visualize Lpf fimbrial structures and the lack of antibodies specific to Lpf. Our results (summarized in Fig. 5) demonstrate an important role of Lpf in EHEC pathogenicity and strengthen the hypothesis that, in the initial stages of colonization, EHEC preferentially infect the ileal region and subsequently colonize the large intestinal mucosa. We also demonstrated that Lpf are needed to target M-cells in small intestinal Peyer’s patches. A better understanding of Lpf-related events in the human gut, as provided in the present study, will be helpful for the development of early intervention strategy against EHEC infections.

## Material and Methods

### Bacterial strains, plasmids and culture conditions

Bacterial strains and plasmids used in this study are listed in Table 1. A collection of 236 STEC strains (for which the serotype and seropathotypes were previously described^26^,^27^) was tested for the presence of *lpf* genes. Bacteria were grown overnight at 37 °C without shaking in LB broth, or DMEM medium (PAA) supplemented with 10% fetal bovine serum (FBS) (Lonza). When required, appropriate antibiotics were added to the media at the following final concentrations: kanamycin (PAA) 50 μg/ml, ampicillin (PAA) 50 μg/ml and chloramphenicol (PAA) 25 μg/ml. L-Arabinose (Sigma-Aldrich) was used at a final concentration of 10 mM with trans-complemented mutants to promote induction of *lpf* genes cloned into the pBAD24 and pBAD33 vectors.

### Prevalence of genes encoding *lpf*_*OI-141*_, *lpf*_*OI-154*_ and *lpf*_*OI-113*_ in STEC strains

All STEC isolates were analyzed for the presence of genes encoding for Lpf_OI-141_, Lpf_OI-154_ and Lpf_OI-113_. The corresponding *lpf*_*OI-141*_, *lpf*_*OI-154*_ and *lpf*_*OI-113*_genes were amplified by PCR using the primers listed in Supplemental Table 1.

### Survival and expression of *lpf* genes under human simulated digestive conditions

#### Batch cultures with bile salts

EHEC O157:H7 EDL933 was grown without shaking at 37 °C in LB or DMEM medium (PAA), supplemented or not with 2% bile salts (50% cholic acid sodium salt and 50% deoxycholic acid sodium salt; Sigma) until exponential (3 h) or stationary growth phase. Samples were collected to determine *lpf* expression. Three independent experiments were performed.

#### TIM Gastric and Small Intestinal System

Human upper digestive tract conditions were reproduced by the dynamic TNO GastroIntestinal model (TIM, TNO) in which four serial compartments simulate the stomach and the three segments of the small intestine, *i.e.* duodenum, jejunum, and ileum^67^. The main parameters of human digestion, are reproduced as accurately as possible based upon *in vivo* data (Supplemental Table 2). TIM was programmed to reproduce the GI conditions of a healthy adult after intake of a glass of water (Supplemental Table 2). Mineral water (200 ml) was experimentally inoculated with EHEC O157:H7 strain EDL933 (10^7 ^CFU/ml). Two types of *in vitro* digestions were performed: gastric digestions where only the gastric compartment was set-up (total duration of 60 min) and GI digestions using the entire TIM model (total duration of 300 min). Samples were taken in the initial bacterial suspension (t0) and regularly collected in the different compartments during GI digestions to determine EHEC survival. Gastric and ileal effluents were kept on ice and pooled on 0–10, 10–20, 20–40 and 40–60 min for gastric digestion and hour-by-hour for GI digestion. Samples were taken in each fraction and stored at −80 °C until RNA extraction for the determination of *lpf* expression. Four independent experiments were performed.

#### ARCOL large intestinal model

Human colonic conditions were simulated in the ARtificial COLon (ARCOL). ARCOL is a one-stage fermentation system used under semi-continuous conditions (Applikon). The ARCOL model was set-up to mimic the average conditions found in the colon of a healthy human adult (Supplemental Table 2). The bioreactor was inoculated with fresh feces collected from a healthy individual with no history of antibiotic treatment 3 months before the study. EHEC O157:H7 strain EDL933 was inoculated in the bioreactor after a 4-day stabilization period (10^7 ^CFU/ml). Fermentations were carried out in triplicate using fresh feces collected from three adult volunteers. Samples were regularly collected from the colonic medium to determine the survival kinetic of the pathogen and *lpf* expression.

#### EHEC survival

EDL933 survival in the TIM (gastric and ileal compartments) and in the ARCOL models was determined by direct plating onto LB agar and qPCR analysis using *stx1* specific primers (Supplemental Table 1)^42^. Results were expressed as percentages of initial intake and cross-compared to those obtained with a theoretical non-absorbable transit marker, blue dextran, giving a 100% survival rate for bacteria. The concentrations of blue dextran used as a transit marker in the TIM system were determined colorimetrically using a spectrophotometer(DU^®^640 B Spectrophotometer, Beckman Coulter, Ville-pinte, France) at λ = 595 nm. In the ARCOL model, survival rates were assessed by comparing the profiles obtained for bacteria with that of a mathematical theoretical marker that simulates the behavior of an inert (i.e., non-degraded and non-absorbed) compound. Its removal from the bioreactor was described by the formula : *C*_*t*_ = *C*_0_ × e^(−*t*/τ)^, where *C*_*t*_ is the concentration of the marker at time *t, C*_0_ its initial concentration, *t* the time of fermentation, and τ the residence time^68^. Bacterial counts below that of the transit marker reflect cell mortality, while counts above the transit marker are indicative of bacterial growth.

#### Expression of *lpf* genes

Total RNA was extracted from batch cultures and digestive samples (gastric and ileal effluents and colonic medium) using TRIzol^®^ reagent method adapted from Toledo-Arana *et al*.^69^. RNAs were reversely transcribed using the First-Strand cDNA synthesis kit (Takara) and qRT-PCR was performed using SYBR Green qPCR Master Mix (Roche) on a Biorad qPCR system with specific primers listed in Supplemental Table 1. *Enterobacteriaceae* 16 S was used as internal control for quantification of mRNA expression. Fold induction was calculated using Ct method as follows:


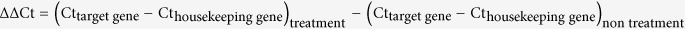


and the final data were derived from 2^−ΔΔCt^.

### Characterization of type I pili expression in EHEC O157:H7 EDL933

#### Preparation of fimbrial crude extracts

EHEC O157:H7 EDL933 and AIEC LF82 (as a positive control) were grown for 3 h in DMEM (PAA) with 2% of bile salts. Detailed step-by-step protocols for pili extraction and immunoblotting analysis can be found in the Supplemental Methods.

#### Transmission electron microscopy

Type I pili and bacterial integrity of *lpf* mutants were visualized by transmission electron microscopy (TEM). EHEC O157:H7 strain EDL933 and AIEC strain LF82 (as a positive control) were grown overnight in LB broth at 37 °C without shaking. A drop of the culture was placed for 2 min on carbon-Formvar copper grids (Electron Microscopy Sciences, Hatfield, England) and negatively stained during 30 s with acid phosphotungstic pH 6.0. Grids were examined with Hitachi H-7650 transmission electron microscope.

### Construction and characterization of *lpf* mutants

#### Construction of isogenic mutants and transcomplementation

Isogenic mutants of strain EDL933 deleted of *lpfA*_*OI-141*_(*lpfA* from O island 141) and/or *lpfA*_*OI-154*_(*lpfA* from O island 154) were generated using the PCR method described by Datsenko and Wanners^70^ and modified by Chaveroche *et al*.^71^ (see details in Supplemental Methods).

#### Bacterial growth kinetics and motility assay

Bacterial strains (wild-type EDL933, *lpf* isogenic mutants, trans-complemented strains, and O6:H10 strain NV110, naturally deficient in Lpf^33^,^34^) were grown overnight at 37 °C without shaking on LB broth. Bacterial growth was monitored for 12 h. For mobility assays, 0.5 μl of the overnight cultures were inoculated in the middle of 0.3% LB agar plates. After incubation (24 h, 37 °C), motility was assessed qualitatively by examining the circular swim formed by the growing motile bacterial cells. The EDL933-*ΔfliC* isogenic mutant was used as a negative control.

#### Shiga toxin production

To quantify Stx production, bacterial supernatant of an overnight culture in LB broth were tested for cytotoxicity in the Vero cell assay, as previously described^15^,^34^ (see details in Supplemental Methods).

### Role of Lpf in the interactions of EHEC strains with the intestinal epithelium

#### Adhesion assays

The human colorectal adenocarcinoma cell-line Caco-2 (ATCC HTB37) was grown at 37 °C under 5% CO_2_ in complete DMEM (PAA) supplemented with 10% heat-inactivated fetal bovine serum (FBS) (Lonza), 4 mM L-glutamine (PAA), 100 U/ml penicillin (PAA) and 100 mg/ml streptomycin (PAA). All bacterial strains (wild-type EDL933, *lpf* isogenic mutants, trans-complemented mutants and O6:H10 strain NV110) were incubated for 3 h at 37 °C in DMEM (PAA) in the presence of 2% bile salt before cell infection. *E. coli* K12-C600 and AIEC LF82 were used as negative and positive controls, respectively. Each experiment was performed as described previously^30^. Briefly, after three washes with PBS, Caco-2 cells were infected with bacteria grown for 3 h at 37 °C at a multiplicity of infection (MOI) of 100. After 3 h, non-adherent bacteria were removed from the cells by three washes with PBS. To quantify the number of bacteria adherent to epithelial cells, the cells were scraped with 1% triton X-100 (Sigma) in PBS, and serial 10 fold dilutions were plated overnight at 37 °C onto LB agar plates.

#### Translocation across M-cell monolayers

The *in vitro* M-cell co-culture model was first developed by Kerneis *et al*.^72^ and later adapted by Gullberg *et al*.^73^. The human colorectal adenocarcinoma cell-line Caco-2 cl1^73^ was grown as previously described for the adhesion assays. The human Burkitt’s lymphoma cell-line Raji B (ECACC 85011429) was grown in complete RPMI-1640 medium (PAA) supplemented with 10% heat-inactivated FBS, 8 mM L-glutamine (PAA), 100 U/ml penicillin (PAA) and 100 mg/ml streptomycin (PAA). A total of 1.10^6^ Caco-2 cl1 cells per ml were seeded onto the apical aspect of Transwell^TM^ filters (Millipore Ltd) previously coated with BD Matrigel^TM^. Cells were carefully cultured for 17 days until they reached a fully differentiated phenotype. Then 5∙10^5^ Raji-B cells were added to the basolateral compartment of Caco-2 cl1 monolayers, and co-culture was maintained for 4–6 days. Monocultures of Caco-2 cl1 cells on matched filter supports were used as control. For translocation assays, apical surface of M-cells or Caco-2 cl1 were infected with 1∙10^7^ EHEC bacteria per Transwell^TM^. Samples from basolateral media were collected every 2 h for 6 h and 10-fold dilutions were plated onto LB agar. The integrity of cell monolayers was tested by monitoring trans-epithelial electrical resistance (TEER) with a Millicell^®^-ERS (Millipore). Each experiment was performed in triplicate.

#### Ethics statement

Work on animals was performed in compliance with French and European regulations on care and protection of laboratory animals (EC Directive 2010/63, French Law 2013–118, February 6, 2013). All experiments were approved by the Comité d’Ethique en Matière d’Expérimentation Animale Auvergne (CEMEAA), registered under the reference C2EA-02. All efforts were made to minimize animal suffering.

#### Animals

Adult (5–6 weeks old) male FVB mice were purchased from Charles River Laboratories. Animals were housed in the Université d’Auvergne Medical School animal facility accredited by the French Ministry of Agriculture for performing experiments on live rodents (Agreement 2014 C63 113 15).

#### Mice ileal loop assay

*In vivo* interactions of EHEC bacteria with Peyer’s patches were studied using mouse ileal loops, as previously described by Hitotsubashi *et al*.^74^. Briefly, mice (n = 10) were starved 24 h before operation, anesthetized and the abdominal cavity was exteriorized through a midline incision. A 6 cm ileal segment with two to three Peyer’s patches was isolated and ligated, and then inoculated by mixed inoculi comprising equivalent numbers (5∙10^8 ^CFU/ml) of the EDL933 wild-type strain in combination with either *lpf* isogenic mutants, transcomplemented mutants or the O6:H10 strain (input). Five hours after injection, mice were euthanized by cervical dislocation according to animal care procedure. Ileal mucosa with or without Peyer’s patches were treated with gentamicin 20 μg/ml for 1 h. Intestinal samples were then crushed with an Ultra-Turrax in the presence of 0.1% Triton X-100 and the number of intracellular bacteria was determined by plating on appropriate selective media (output). Competitive index (CI) analysis was performed to provide a sensitive measurement of the relative degree of attenuation^75^. CI is defined as the ratio of the tested strain (mutant, trans-complemented or O6:H10) to O157:H7 wild-type strain in the output, divided by the ratio of the two strains in the input. Each Peyer’s patch was also macroscopically examined to determine its hemorrhagic status.

### Statistical analysis

During TIM and ARCOL experiments, significant differences in survival between time points were tested using a non-parametric analysis of repeated measures with the “f1.ld.f1” function of the R package “nparLD”^76^ in R 3.2.4^77^. In case of significant difference compared to the transit marker, the function “npar.t.test” of the package “nparcomp”^78^ was used for each time point. In case of a significant interaction effect, a linear mixed effect models with a random intercept on experiments to take account of repeated measures was performed and followed by function “difflsmeans” of the package “lmerTest”^79^. The kinetics of *lpf* genes expression in the TIM and ARCOL models were tested with the “ld.f1” function of the R package “nparLD”. In case of a significant time effect, pairwise comparisons with Bonferroni adjustment were performed. The significance of differences in the ability of adhesion between the bacteria strains and for *lpf* expression in batch cultures were tested using Dunnett’s test. For experiments with multiple treatment groups (*in vitro* M-cell assays), a non-parametric assay of repeated measures was performed, followed by Tukey’s pairwise comparisons. Significance differences for competitive index in *in vivo* ileal loops assays was done by nonparametric Wilcoxon signed rank test. The independence between treatment and hemorrhagic status of Peyer’s patches was tested by chi-square with Yates correction.

## Additional Information

**How to cite this article:** Cordonnier, C. *et al*. Enterohemorrhagic *Escherichia coli* pathogenesis: role of Long polar fimbriae in Peyer’s patches interactions. *Sci. Rep.* **7**, 44655; doi: 10.1038/srep44655 (2017).

**Publisher's note:** Springer Nature remains neutral with regard to jurisdictional claims in published maps and institutional affiliations.

## Supplementary Material

Supplementary Information

## Figures and Tables

**Table 1:** Bacterial strains and plasmids.

Strains or plasmids	Relevant characteristic (s)	*stx* genotype	Source or reference
Strains			
O157:H7	Enterohemorragic *E. coli* O157:H7 reference strain EDL933	*stx1* + *stx2*+	ATCC 43895
O157:H7-*ΔlpfA*_*OI-141*_	EDL933 isogenic mutant with *lpfA*_*OI-141*_ gene deleted, Km^R^	*stx1* + *stx2*+	Mutant generated in this study
O157:H7-*ΔlpfA*_*OI-154*_	EDL933 isogenic mutant with *lpfA*_*OI-154*_ gene deleted, Km^R^	*stx1* + *stx2*+	Mutant generated in this study
O157:H7-*ΔlpfA*_*OI-141*_/*ΔlpfA*_*OI-154*_	EDL933 isogenic mutant with *lpfA*_*OI-141*_ and *lpfA*_*OI-154*_ genes deleted, Km^R^	*stx1* + *stx2*+	Mutant generated in this study
O157:H7-*Δstx2*	EDL933 isogenic mutant with *stx2* gene deleted, Km^R^	*stx1* + *stx2*−	80
O157:H7-*ΔfliC*	EDL933 isogenic mutant with flagellar gene *fliC* deleted, Km^R^	*stx1* + *stx2*+	80
O6:H10	Enterohemorragic *E. coli* O6:H10 strain NV110, naturally lacking *lpf* genes	*stx1* − *stx2*+	29,33
STEC collection	236 STEC strains isolated from cattle, food and patients	NA	29,30
K-12 C600	Non pathogenic *E. coli*	NA	Laboratory
Plasmids			
pKOBEG	pBAD cloning vector harboring λ phage redγβα operon, Cm^R^	NA	70
pBAD24	Cloning vector with arabinose inducible promoter, Amp^R^	NA	70
pBAD33	Cloning vector with arabinose inducible promoter, Cm^R^	NA	70
pBAD24-*lpfA*_*OI-141*_	pBAD24 harboring the 537-bp NcoI-PstI fragment containing the entire *lpfA*_*OI-141*_ gene	NA	Plasmid generated in this study
pBAD33-*lpfA*_*OI-154*_	pBAD33 harboring the 603-bp HindIII-XbaI fragment containing the entire *lpfA*_*OI-154*_ gene	NA	Plasmid generated in this study

NA, not applicable; *lpfA*_*OI-141*_*, long polar fimbrae,* subunit A from O island 141*; lpfA*_*OI-154*_*, long polar fimbrae,* subunit A from O island 154*; stx1*, Shiga-toxin 1; *stx2*, Shiga-toxin 2; ^R^antibiotic resistant mutant; Km, kanamycin; Amp, ampicillin; Cm, chloramphenicol.
