# Supplementary material for: Enterohemorrhagic Escherichia coli pathogenesis: role of Long polar fimbriae in Peyer’s patches interactions
Source: Sci Rep. 2017 Mar 20;7:44655. doi: 10.1038/srep44655 (PMC5357955; doi:10.1038/srep44655)
Supplement: Supplementary Information [file srep44655-s1.pdf]

Supplementary informations

Enterohemorrhagic *Escherichia coli* pathogenesis*:* role of Long Polar Fimbriae in Peyer's patches interactions

Charlotte Cordonnier[1,2], Lucie Etienne-Mesmin[1,2,3], Jonathan Thévenot[1,2,4], Amandine Rougeron[1], Sandra Rénier[1], Benoit Chassaing[1,3], Arlette Darfeuille-Michaud[1†], Nicolas Barnich[1], Stéphanie Blanquet-Diot[2], Valérie Livrelli[1,5]*

[†]In memoriam

[1]Université Clermont Auvergne, Inserm U1071, M2iSH "Microbes, Intestin, Inflammation et Susceptibilité de l'Hôte", USC-INRA 2018, F-63000 Clermont-Ferrand, France

[2]Université Clermont Auvergne, MEDIS " Microbiologie Environnement DIgestif Santé" », F-63000 Clermont-Ferrand, France

[3]current address: Institute for Biomedical Sciences, Georgia State University, Atlanta, GA, 30303, USA

[4]current address: STLO, "Science et Technologie du Lait et de l'Oeuf " Agrocampus Ouest, INRA, Rennes, 35042, France

[5]CHU Clermont-Ferrand, Service de Bactériologie, Parasitologie Mycologie, Clermont-Ferrand, 63000, France

[*]Corresponding author E-mail: valerie.livrelli@udamail.fr (VL)

Supplemental Methods

Detection of promoter deletion in *fim* operon. The 16 bp deletion in the *fim* promoter resulting in the absence of expression of type I pili was investigated by PCR as previously described by Shaikh *et al.*[1]. Primers A and B (Supplemental Table 1) produce a 952 bp amplicon if the deletion is absent, but no amplicon in the presence of the deletion. Primers C and B (Supplemental Table 1) produce a 936 bp amplicon if the deletion is present in the *fim* operon.

Preparation of fimbrial crude extracts. EHEC O157:H7 EDL933 and AIEC LF82 (as a positive control) were grown for 3 h in DMEM (PAA) with 2% of bile salts. After centrifugation (10,000 g, 10 min, 4°C), bacterial pellets were washed in 5 mM Tris 5% NaCl, then centrifuged using same conditions. Pellets were suspended in 5 mM Tris and bacterial surface proteins were sheared off with a homogenizer set at low speed during 2 min (on ice, 3 times). Bacterial cells and debris were sedimented (10,000 g, 10 min, 4°C), while the supernatants constituting the crude extract of bacterial surface proteins were centrifuged (27,000 g, 30 min, 4°C). Pili extracts were obtained as a pellet after ultracentrifugation (270,000 g, 2 h, 4°C). After solubilization in 5 mM Tris buffer (overnight, 4°C), fimbriae were precipitated with 0.1 M $MgCl_2$ (3 h, 4°C). Suspensions were centrifuged (27,000 g, 40 min, 4°C) and supernatants were discarded. Fimbriae were solubilized in PBS then subjected to depolymerization by trifluoroacetic acid (TFA) hydrolysis. Samples were acidified to pH 2 with final 0.16% TFA, heated at 100°C for 5 min, cooled and neutralized with NaOH before immunoblotting analysis

Immunoblotting. Fimbrial crude extracts were incubated with 4X Laemmli buffer containing 10% mercaptoethanol (3 min, 100°C), then resolved on a 12% SDS-PAGE followed by electroblotting onto nitrocellulose membrane (GE Healthcare Life Sciences) using a Trans-Blot® Turbo™ Blotting System (Bio-Rad). One percent of bovine serum albumin (BSA) in TBS-T (10 mM Tris-HCl pH 7.5, 150 mM NaCl, 0.05% Tween 20) was used for diluting antibodies and blocking non-specific binding to nitrocellulose membrane. Type I pili were detected by Western blotting using the primary anti-pili polyclonal antibody αF1 from rabbit (1:5000) and the secondary monoclonal HRP-conjugated anti-rabbit IgG from goat (1:5000). The blot was developed using enhanced chemiluminescence reagent (Bio-Rad).

Yeast agglutination assay. EHEC O157:H7 strain EDL933 and AIEC strain LF82 (as a positive control) were cultured in static LB broth at 37°C overnight. Bacterial cells were collected by centrifugation (5,000 g, 10 min, room temperature) and pellets were re-suspended in 100 µl of 1X PBS. Subsequently, 50 µl of a 2% (vol/vol) suspension of *Saccharomyces cerevisiae* (Sigma) and an equal amount of undiluted or serial dilutions (from ½ to 1/20) of bacterial cells were mixed in 96-well plate. After 1 h incubation, visible agglutination was determined macroscopically.

Construction of isogenic mutants and transcomplementation. For double mutant Δ$lpfA_{OI\text{-}141}$/Δ$lpfA_{OI\text{-}154}$ generation, kanamycin cassette was excised using pcp20 plasmid after the first gene deletion[2]. Primers used are listed in Supplemental Table 1. For transcomplementation assays, PCR products containing the entire major fimbrial subunit $lpfA_{OI\text{-}141}$ or $lpfA_{OI\text{-}154}$ were cloned into the pBAD24 and pBAD33 vectors, respectively, and introduced in their respective mutant strain as previously described[3].

Shiga toxin production. EDL933-Δ*stx2* was used as a negative control. The Stx titre was expressed as the reciprocal of the highest filtrate dilution that cause 50% cell detachment after 24 h incubation, as judged by the dye density and by microscopic observation. Purified Stx2 (Toxin Technology) was used to estimate the range of toxin production. Each experiment was performed three times.

Supplemental References

1. Shaikh, N., Holt, N. J., Johnson, J. R. & Tarr, P. I. Fim operon variation in the emergence of Enterohemorrhagic Escherichia coli: an evolutionary and functional analysis. *FEMS Microbiol. Lett.* **273,** 58–63 (2007).
2. Datsenko, K. A. & Wanner, B. L. One-step inactivation of chromosomal genes in Escherichia coli K-12 using PCR products. *Proc. Natl. Acad. Sci. U. S. A.* **97,** 6640–6645 (2000).
3. Guzman, L. M., Belin, D., Carson, M. J. & Beckwith, J. Tight regulation, modulation, and high-level expression by vectors containing the arabinose PBAD promoter. *J. Bacteriol.* **177,** 4121–4130 (1995).

Supplemental Figures

A

Origin	Total no. of strains (%)	No. of lpf-positive strains (%)									
		lpf_{OI-141}^{+}	lpf_{OI-154}^{+}	lpf_{OI-113}^{+}	lpf_{OI-141}^{+} only	lpf_{OI-154}^{+} only	lpf_{OI-113}^{+} only	lpf_{OI-141}^{+}/ lpf_{OI-154}^{+}	lpf_{OI-141}^{+}/ lpf_{OI-113}^{+}	lpf_{OI-154}^{+}/ lpf_{OI-113}^{+}	lpf_{OI-141}^{+}/ lpf_{OI-154}^{+}/ lpf_{OI-113}^{+}
**HUS and HC**	27 (11.4)	9 (3.8)	21 (8.9)	8 (3.4)	0 (0)	9 (3.8)	4 (1.7)	9 (3.4)	0 (0)	3 (1.3)	1 (0.4)
**Asymptomatic children**	10 (4.2)	0 (0)	0 (0) $^{\beta}$	6 (2.5)	0 (0)	0 (0)	6 (2.5)	0 (0)	0 (0)	0 (0)	0 (0)
**Food**	24 (10.2)	3 (1.3)	3 (1.3) $^{\beta}$	17 (7.2) $^{\alpha}$	0 (0)	0 (0)	16 (6.8)	2 (0.8)	0 (0)	0 (0)	1 (0.4)
**Bovine feces**	175 (74.2)	3 (1.3) $^{\beta}$	6 (2.5) $^{\beta}$	112 (47.5) $^{\alpha}$	0 (0)	0 (0)	109 (46.2)	3 (1.3)	0 (0)	3 (1.3)	0 (0)
**TOTAL**	**236 (100)**	**15 (6.4)**	**30 (12.7)**	**143 (60.6)**	**0 (0)**	**9 (3.8)**	**135 (57.2)**	**13 (5.5)**	**0 (0)**	**6 (2.5)**	**2 (0.8)**

B

Seropathotype	Total no. of strains (%)	No. of lpf-positive strains (%)									
		lpf_{OI-141}^{+}	lpf_{OI-154}^{+}	lpf_{OI-113}^{+}	lpf_{OI-141}^{+} only	lpf_{OI-154}^{+} only	lpf_{OI-113}^{+} only	lpf_{OI-141}^{+}/ lpf_{OI-154}^{+}	lpf_{OI-141}^{+}/ lpf_{OI-113}^{+}	lpf_{OI-154}^{+}/ lpf_{OI-113}^{+}	lpf_{OI-141}^{+}/ lpf_{OI-154}^{+}/ lpf_{OI-113}^{+}
**A**	11 (4.7)	11 (4.7)	11 (4.7)	0 (0)	0 (0)	0 (0)	0 (0)	11 (4.7)	0 (0)	0 (0)	0 (0)
**B**	12 (5.1)	0 (0) $^{\alpha}$	9 (3.8)	1 (0.4)	0 (0)	9 (3.8)	1 (0.4)	0 (0)	0 (0)	0 (0)	0 (0)
**C**	73 (30.9)	1 (0.4) $^{\alpha}$	6 (2.5) $^{\alpha}$	64 (27.1) $^{\alpha}$	0 (0)	0 (0)	58 (24.6)	0 (0)	0 (0)	5 (2.1)	1 (0.4)
**D**	50 (21.2)	1 (0.4) $^{\alpha}$	1 (0.4) $^{\alpha}$	26 (11) $^{\beta}$	0 (0)	0 (0)	26 (11)	1 (0.4)	0 (0)	0 (0)	0 (0)
**E**	90 (38.1)	2 (0.8) $^{\alpha}$	3 (1.3) $^{\alpha}$	52 (22) $^{\alpha}$	0 (0)	0 (0)	50 (21.2)	1 (0.4)	0 (0)	1 (0.4)	1 (0.4)
**TOTAL**	**236 (100)**	**15 (6.4)**	**30 (12.7)**	**143 (60.6)**	**0 (0)**	**9 (3.8)**	**135 (57.2)**	**13 (5.5)**	**0 (0)**	**6 (2.5)**	**2 (0.8)**

Supplemental Figure 1. The presence of $lpf_{\text{OI-141}}$, $lpf_{\text{OI-154}}$ and $lpf_{\text{OI-113}}$ operons was determined by PCR in 236 STEC/EHEC isolates and allocated according to the strain source **(A)** and seropathotype **(B)**. Results are expressed as total number of *lpf*-positive strains and as percentages of total strains (in bracket). Prevalence of each *lpf* operon in the seropathotype A was compared to that in other seropathotypes and statistical differences were indicated by α ($p < 0.01$) or β ($p < 0.001$). Prevalence of *lpf* genes was determined and expressed as percentages of the total number of strains in each seropathotype.

A B

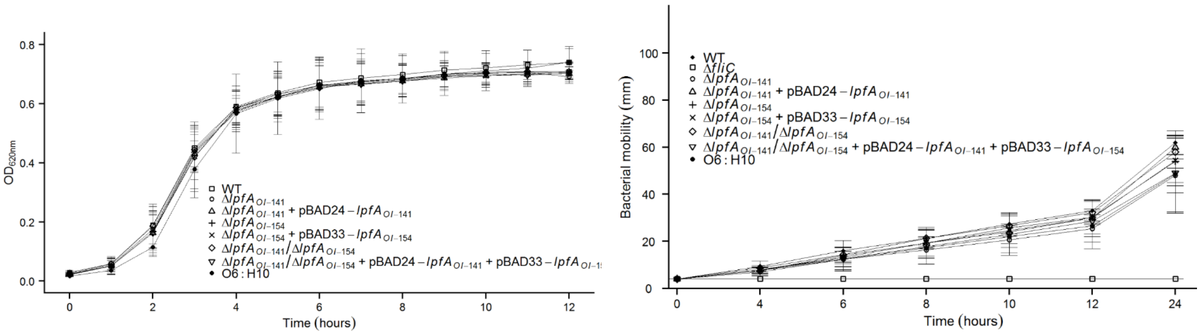

C

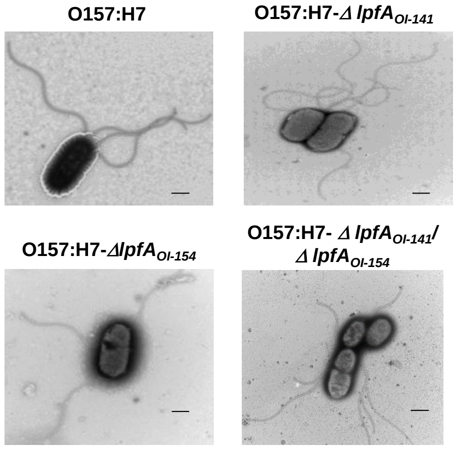

D

Strains	Cytotoxic titer	Stx concentration (ng/mL)
WT O157:H7 strain EDL933	1/256	8.2 ± 1.8 ng/ml
O157:H7-*Δstx2*	-	-
O157:H7-*ΔlpfA*$_{OI-141}$	1/256	8.2 ± 1.8 ng/ml
O157:H7-*ΔlpfA*$_{OI-141}$ + pBAD24-*lpfA*$_{OI-141}$	1/256	8.2 ± 1.8 ng/ml
O157:H7-*ΔlpfA*$_{OI-154}$	1/256	8.2 ± 1.8 ng/ml
O157:H7-*ΔlpfA*$_{OI-154}$ + pBAD33-*lpfA*$_{OI-154}$	1/256	8.2 ± 1.8 ng/ml
O157:H7-*ΔlpfA*$_{OI-141}$/*ΔlpfA*$_{OI-154}$	1/256	8.2 ± 1.8 ng/ml
O157:H7-*ΔlpfA*$_{OI-141}$/*ΔlpfA*$_{OI-154}$ + pBAD24-*lpfA*$_{OI-141}$ + pBAD33-*lpfA*$_{OI-154}$	1/256	8.2 ± 1.8 ng/ml
O6:H10 strain NV110	1/256	8.2 ± 1.8 ng/ml

E

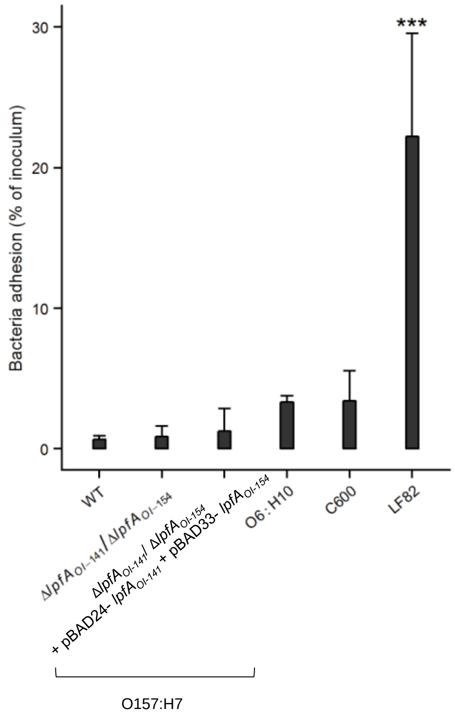

Supplemental Figure 2. Bacterial growth, mobility, cytotoxicity and EHEC EDL933 adhesion to intestinal epithelial cells are not influenced by *lfp* deletion. (A) Growth kinetic in LB broth of wild-type EHEC O157:H7 EDL933, *lpf* isogenic mutants, trans-complemented

and O6:H10 NV110 strains was analyzed by measurement of optical density at 620 nm. Results are expressed as means ± standard deviations (n = 3). **(B)** Bacterial mobility was assessed on 0.3% LB agar plates (24 h, 37°C) by visualization of halo radial diffusion around the primary inoculum. O157:H7 EDL933-Δ*fliC* was used as a negative control. Results are expressed as means ± standard deviations (n = 3). **(C)** Transmission electron micrographs of negatively stained wild-type O157:H7 strain EDL933 and $\Delta lpfA_{OI\text{-}141}$, $\Delta lpfA_{OI\text{-}154}$ and $\Delta lpfA_{OI\text{-}141}$ / $\Delta lpfA_{OI\text{-}154}$ isogenic mutants at × 15,000 magnification (scale bars = 0.5 µm). **(D)** Quantification of Stx production using a Vero toxin assay. Results are expressed as the reciprocal of the highest sample dilution causing 50% cell detachment after 24 h incubation and as the corresponding Stx concentration in ng/ml ± standard deviations (n = 3). No cytotoxic effect (-). **(E)** Caco-2 cells were infected at MOI 100 with EHEC bacteria (wild-type EHEC O157:H7 strain EDL933, *lpf* isogenic mutants, trans-complemented strains and O6:H10 strain NV110) after a 3 h growth in DMEM supplemented with 2% bile salts. *E. coli* C600 and AIEC LF82 were used as negative and positive controls, respectively. The number of adherent bacteria was determined by plating 3 h after infection. Results are given as mean percentages of infective dose ± standard deviations (n = 4). Statistically different from EDL933 wild-type strain at p < 0.001 (***).

Primer name	Oligonucleotide sequence (5' to 3')	Use	Reference
**Prevalence of *lpfA*$_{OI\text{-}141}$, *lpfA*$_{OI\text{-}154}$ and *lpfA*$_{OI\text{-}113}$ in the strains collection**			
lpfO141-F	CTGCGCATTGCCGTAAC	Prevalence analysis of *lpfA*$_{OI\text{-}141}$	[71]
lpfO141-R	ATTTACAGGCGAGATCGTG		
O154-FCT	GCAGGTCACCTACAGGCGGC	Prevalence analysis of *lpfA*$_{OI\text{-}154}$	[71]
O154-RCT	CTGCGAGTCGGCGTTAGCTG		
lpfA-F	ATGAAGCGTAATATTATAG	Prevalence analysis of *lpfA*$_{OI\text{-}113}$	[71]
lpfA-R	TTATTTCTTATATTCGAC		
**Construction of *lpf* isogenic mutants and transcomplemented strains**			
K1	CAGTCATAGCCGAATACCCT	Kanamycin resistance cassette amplification	[71]
K2	CGGTGCCCTGAATGAACTGC		
Kt	CGGCCACAGTCGATGAATCC		
	ΔlpfA$_{OI\text{-}141}$ **ISOGENIC MUTANT**		
MilpfA1-1	ACTTTGACGACTAATGGCGGCAATTACGCCGCCATTTGTAAAACGGACGAGTAGGC TGGA	EDL933-ΔlpfA$_{OI\text{-}141}$ isogenic mutant construction	This study
MilpfA1-2	AGTTGTCGATTTTAATACATCAAGATTTTCTTTTTAATGTAATTTTTTAACATATGAA TATCC		
lpf A1-1	CGTTGCCAGAGTAGAAACGT	Isogenic mutant verification	This study
lpf A1-2	GAGCTTTAGTGGTGTTGGTG		
lpf A1-3	CTGCCATCATAAATTACGCGA	Isogenic mutant verification	This study
lpf A1-4	GCTATTAATATATTATTCGTATAATT		
NcoI-LpfA1	ATTATTCCATGGAGTTTTTCATGAAAAACGTCG	Transcomplementation construction	This study
PstI-LpfA1	ATTATTCTGCAGTTACTCGTAAGACAGGTTGAAATC		
	ΔlpfA$_{OI\text{-}154}$ **ISOGENIC MUTANT**		
MilpfA2-1	AACCATAATGGCGAGCGCCATTTTATTTAAGGTATTATGTTAAGAATGATGTAGGCT GGAGCT	EDL933-ΔlpfA$_{OI\text{-}154}$ isogenic mutant construction	This study
MilpfA2-2	AATAAATGGATAACACACAGGAATAATATCAAATTAATGGAGTTATATTTCATATG AATATCC		
lpf A2-1	CGAGCCATGAACTTCATGGA	Isogenic mutant verification	This study
lpf A2-2	CTGGATAACTCTAACGATGCT		
lpf A2-3	GTAGCCATCGACATCTGCG	Isogenic mutant verification	This study
lpf A2-4	GACTGTTTTCTACGCGAATTC		
XbaI-LpfA2	AATAATTCTAGAAGGAGGAATTCACCATGAAAACCAAATATGATTGTAGGAGC	Transcomplementation construction	This study
HindIII-LpfA2	ATTATTGCATGCTTATTTTATATATTCAACTGTAAACTGCGAG		
***fim* promoter region polymorphisms**			
Primer A	CTCAAGCATAAAAATTTTAAAAAACGA	Determination of the 16 bp deletion in the *fim* promoter	[71]
Primer B	TAACCTACCCAGGTTCAGGGACGT		
Primer C	CTCAAGCATAAAAATTTTAAACTAACTG		
**Survival kinetic of EHEC in the ARCOL model**			
VT1c	ACCCTGTAACGAAGTTTGCG	Target gene: Shiga-toxine 1	[72]
VT1d	ATCTCATGCGACTACTTGAC		
**Expression of *lpfA*$_{OI\text{-}141}$ and *lpfA*$_{OI\text{-}154}$ and *lpfA*$_{OI\text{-}113}$ in batch cultures and in the TIM and ARCOL models**			
RT-lpfO154-F	TATGGCAGGTCACCTACAGG	Expression analysis of *lpfA*$_{OI\text{-}154}$	This study
RT-lpfO154-R	AGGTTTCCGGGCATTGAGTC		
LPFA1-CF	GGTTGGTGACAAATCCCCG	Expression analysis of *lpfA*$_{OI\text{-}141}$	[72]
LPFA1-CR1	CGTCTGGCCTTTACTCAGA		
Eco1457F	CATTGACGTTACCCGCAGAAGAAGC	Housekeeping gene *Enterobacteriaceae* 16S	[73]
Eco1652R	CTCTACGAGACTCAAGCTTGC		

Supplemental Table 1. Primers Used for PCR, qPCR and RT-qPCR Analyses.

	***In vitro* models**	
	**TIM-1**<br>(gastric and small intestinal model)	**ARCOL**<br>(large intestinal model)
**Main parameters**	Body temperature (37°C)	
	Gastric pH from 6 to 1.7<br>Duoddenal pH 6.4<br>Jejunal pH 6.9<br>Ileal pH 7.2	Colonic pH 6.3
	Chyme mixing	Redox potential (-400 mV)
	Transits time	Retention time (36h)
	Gastric, biliary and pancreatic secretions	Supply of ileal effluents
	Passive absorption of water and digestion products	Passive absorption of water and fermentation metabolites
	No microbiota	Anaerobiosis maintained by resident microbiota activity

Supplemental Table 2. Main parameters of the *in vitro* models TIM-1 and ARCOL
